# The Investigation of Plasma Natriuretic Peptide as Inflammatory Biomarker Predictor in Coronary Heart Disease Patients With Dry Eye Disease: A Case–Control Study

**DOI:** 10.1155/ijin/2197568

**Published:** 2026-09-27

**Authors:** Amani Y. Alhalwani, Ibrahim Alsaggaf, Abdulrahman Alidroos, Farouk W. Kattan, Talal Y. Almarzouki, Sarah Shawli, Maryam Hashem Shawish, Shatha M. Jambi, Jumanah Abdali, Mohammed Fawaz Qutub

**Affiliations:** ^1^ College of Science and Health Professions, King Saud Bin Abdulaziz University for Health Sciences, Jeddah, Saudi Arabia, ksau-hs.edu.sa; ^2^ Department of Biomedical Research, King Abdullah International Medical Research Center, Jeddah, Saudi Arabia, kaimrc.med.sa; ^3^ College of Medicine, King Saud Bin Abdulaziz University for Health Sciences, Jeddah, Saudi Arabia, ksau-hs.edu.sa; ^4^ Faculty of Medicine, King Abdul Aziz University, Jeddah, Saudi Arabia, kau.edu.sa; ^5^ Ibn Sina National College for Medical Studies, Jeddah, Saudi Arabia, ibnsina.edu.sa; ^6^ School of Medicine, Arabian Gulf University, Manama, Bahrain, agu.edu.bh; ^7^ College of Applied Medical Sciences, King Saud Bin Abdulaziz University for Health Sciences, Jeddah, Saudi Arabia, ksau-hs.edu.sa; ^8^ Department of Ophthalmology, Ministry of the National Guard - Health Affairs, Jeddah, Saudi Arabia

**Keywords:** albumin, coronary heart disease, C-reactive protein, dry eye disease, inflammation, natriuretic peptide

## Abstract

**Background:**

Inflammation contributes to both coronary heart disease (CHD) and dry eye disease (DED). Recognizing the relationship between CHD and DED is critical for maintaining ocular health in CHD patients. As a result, early monitoring of natriuretic peptides and inflammatory biomarkers may help minimize DED in CHD patients. This study aims to investigate brain‐type natriuretic peptide and inflammatory biomarkers in blood in CHD–DED.

**Methods:**

This is a retrospective case–control study among CHD–DED patients at the cardiac outpatient clinic of King Abdulaziz Medical City (Jeddah, Saudi Arabia) from January 2016 to June 2024. The study extracted demographic information and diagnostic values of brain natriuretic peptide (BNP), C‐reactive protein (CRP), albumin (Alb), total protein (TP), glycated hemoglobin A1C (HbA1C), troponin I, and creatine kinase (CK) from the electronic medical records of two patient groups: case group; CHD–DED, and control group; CHD only. Statistical analyses were performed via GraphPad Prism. Continuous variables were summarized with appropriate nonparametric descriptive statistics, while categorical variables were reported as frequencies and percentages. Spearman’s correlation analyses were conducted to evaluate the associations among BNP, Alb, TP, and CRP within each group. Partial Spearman’s correlations were performed using regression residuals to control for potential confounding variables, specifically age and body mass index (BMI).

**Results:**

BMI differed significantly between the CHD and CHD–DED groups (*p* < 0.001). There were significant differences in BNP levels among the groups (*p* value = 0.013). In the CHD–DED group, BNP showed a weak, nonsignificant positive correlation with CRP (*r* = 0.21, *p* value = 0.08). Conversely, a significant inverse association was noted between BNP and Alb (*r* = −0.48, *p* value < 0.001). The inverse relationship between BNP and Alb remained statistically significant, even after adjusting for age and BMI, in contrast to the weakened associations observed between BNP and CRP.

**Conclusions:**

This study forecasts the influence of inflammatory biomarker levels of BNP, CRP, Alb, and TP on the correlation strength in patients with CHD–DED, proposing predictors for identifying DED complications in individuals with CHD. Future research into the role of BNP could elucidate its involvement in other inflammatory conditions.

## 1. Introduction

Dry eye disease (DED) is a condition based on the irregularity of the ocular surface and tear stability [1]. DED is a relatively underexamined disease compared to other diseases of the same severity and impact [1]. It has been shown that one in every four ophthalmology patients complain of DED [2]. The prevalence of DED patients across Saudi Arabia was 38.4%, which is high‐risk population including elderly [3]. According to the study, the prevalence of DED varies from moderate to severe among the global population [4]. Additionally, compared to the quality of life of other diseases using a utility assessment, mild DED scored 0.81 (comparable to psoriasis), while severe DED scored 0.72 (equivalent to Class IV angina) [1]. DED has been divided into two tear film categories: tear aqueous‐deficient and tear hyperevaporative [5]. DED has many risk factors, including environmental and systemic factors, that may result in damage to the ocular surface [6]. In addition, aging and systemic disease induce inflammation and make tear production insufficient [7]. DED results from tear film disturbance through several mechanisms, including the dysfunction of meibomian glands that are responsible for secreting Meibum, a substance found in the lipid layer of tears [8]. Meibum preserves the integrity of the tear film, reducing tear evaporation and preventing eye dryness and irritation [9]. Additionally, the condition may indicate a more significant underlying systemic problem related to lipid irregularity, such as coronary heart disease (CHD) [10].

CHD is a multifactorial cardiovascular condition primarily driven by chronic inflammation, which initiates plaque development and is associated with conditions like DED [11]. CHD can exert systemic inflammatory effects that exacerbate or adversely affect the function of other organs [12]. Chronic systemic inflammation is characterized by the sustained release of vital chemical mediators, for instance, C‐reactive protein (CRP); which is extend beyond local inflammation sites, and exert influences on distant organs and tissues, including ocular surfaces [13]. Studies show that CRP substantially and independently predicts unfavorable cardiovascular events (https://paperpile.com/c/F3FGlu/O5HJo) [14]. However, hs‐CRP and other systemic inflammatory biomarkers are associated with DED [15]. Moreover, natriuretic peptides are counterregulatory hormones; they also inhibit neurohormones that cause vasoconstriction and fluid retention and are similarly elevated in heart failure. Brain natriuretic peptide (BNP) is independent of left ventricular ejection fraction and may better predict risk classification following myocardial infarction [16].

Previous study demonstrated that in patients with severe heart disease, elevated BNP levels are linked with microvascular alterations in the eye, as exhibited by thinner central retinal layers and diminished vessel density in particular retinal regions [17]. Additionally, BNP is released from retinal pigment epithelium cells and is expressed in the epithelial components and receptors of retinal ganglion cells within the retina [18, 19]. BNP reported an increase as a marker in hypoxic conditions and also diabetic retinopathy [20, 21]. The BNP correlation in DED patients has not been studied. Furthermore, the risk of coronary artery disease was inversely correlated with serum Alb level [22].

A previous study by Lee et al. reported a possible correlation between the severity of coronary artery disease and the resulting DED [11]. Another study examined data from 12,007 people suffering from DED and 36,021 patients without it, focusing on 33 comorbidity categories including hypertension, ischemic heart disease, and hyperlipidemia [23]. As a result, further research is warranted to determine the connection between DED and cardiovascular disease. The central hypothesis of this research is the investigation of natriuretic peptides and inflammatory blood biomarkers indicators in CHD with DED (CHD–DED) patients. A corollary to our hypothesis is that these blood biomarkers can improve understanding of the pathogenesis, treatment, and prevention of DED in patients with CHD.

## 2. Methodology

### 2.1. Study Population

This study was an exploratory, retrospective case–control design. The demographic and clinical laboratory data were collected from January 2016 to June 2024 through the electronic health records (BestCare) at the outpatient cardiac center and the Cornea Clinic at King Abdulaziz Medical City (KAMC), Ministry of National Guard Health Affairs (MNG‐HA), Jeddah, Saudi Arabia. Patients diagnosed with CHD were determined from cardiology clinic records. The inclusion criteria for selecting the study and control groups were that patients could be of either gender and aged 18 years or older. Patients with other systemic inflammatory diseases, such as rheumatoid arthritis, lupus, scleroderma, Sjogren’s syndrome, and cancer, were excluded based on reported diagnosis in the system. Patients were categorized into two groups based on ophthalmologic clinical assessment and physician examination: CHD without DED (CHD group) and CHD with DED (CHD–DED group).

### 2.2. Patient Sample Size

The actual sample size was 208 patients, divided into two groups: CHD patients (*n* = 140) and CHD–DED patients (*n* = 68) selected based on inclusion and exclusion criteria.

A power analysis for this study group was performed to assess the study’s statistical power using the Clinical Sample Size Calculator on the website [24]. The sample size was determined by the number of study groups and the prevalence of CHD (*n* = 68, 28.7%) [25], resulting in a control group and a prevalence of CHD–DED (*n* = 140, 11%) [26]. To improve statistical power, a 2:1 enrollment ratio was used in the power analysis with a dichotomous primary endpoint and a two‐sided alpha of 0.05. Based on the study group of (Group 1%) in the control group and (Group 2%), the study power was 80%. The calculated sample size was 168, with 56 in the study group and 112 in the control group.

DED patients were selected based on ophthalmology clinical assessment, confirmed by a physician examination, while CHD patients were selected based on the diagnosis from the cardiology clinic. The sample size was determined by the availability of eligible patient data and was deemed adequate for descriptive analysis and exploratory group comparisons. Because this study was exploratory and retrospective, it is limited by the available data in the BestCare system, such as information matched by age, gender, and CHD severity.

### 2.3. Data Collection and Analysis

Patient data were retrospectively collected from the electronic health records (BestCare system), including patient demographics (age, gender, and BMI) and relevant medical history. At the same time, blood biomarker data were gathered from laboratory results taken within a standardized 3‐month period following the confirmed diagnosis of CHD. To maintain temporal consistency, all blood markers analyzed for each patient such as cardiac and stress indicators (BNP, troponin, and total creatine kinase [CK]), inflammatory markers (CRP and glycated hemoglobin A1C [HbA1C]), and nutritional/hepatic markers (Alb, total protein [TP], and aminotransferase [AST]) were obtained from the same clinical visit or lab analysis period.

### 2.4. Statistical Analysis

The statistical analysis was conducted using GraphPad Prism program (GraphPad program Inc., San Diego, CA, USA). The Shapiro–Wilk test was employed to evaluate data normality, finding that continuous variables were not normally distributed (*p* value < 0.05). Continuous variables are presented as median (interquartile range, Q1–Q3) in Tables 1 and 2, while categorical variables are represented as frequencies and percentages. The comparisons between the CHD and CHD–DED groups were conducted using the nonparametric Mann–Whitney U test, which evaluates disparities in data distributions between two independent groups. Spearman’s rank correlation analysis was employed to assess the relationships between inflammatory and protein biomarkers, specifically Alb, TP, CRP, and BNP, within each group. We used regression residuals to conduct partial Spearman’s correlations to control for age and BMI, two potential confounding variables. A *p* value < 0.05 was considered significant.

**TABLE 1:** Demographic data for the CHD and CHD–DED patients.

Parameter	CHD (*n* = 140) Median (Q1–Q3)	CHD–DED (*n* = 68) Median (Q1–Q3)	*p* value^∗^
Age (years)	68 (60–76)	70 (61–76.5)	0.260
BMI (kg/m^2^)	28.38 (24.97–32.87)	30.51 (27.73–34.85)	0.006
Gender, *n* (%)			0.119^∗∗^
Male	96 (68.6%)	41 (60.3%)	
Female	44 (31.4%)	27 (39.7%)	

*Note:* BMI, body mass index (normal range: BMI ≥ 27 kg/m^2^).

^∗^Mann–Whitney test.

^∗∗^ *χ* ^2^ test for categorical variables.

**TABLE 2:** Inflammatory biomarker findings for the CHD and CHD–DED groups.

Inflammatory biomarkers	CHD (*n* = 140) median (Q1–Q3)	CHD–DED (*n* = 68) median (Q1–Q3)	*p* value^∗^
HbA1c	7.90 (7.10–9.40)	7.50 (6.78–8.85)	0.039
CRP	9.40 (3.58–43.88)	11.60 (2.40–49.85)	0.206
TP	70 (66.5–74)	70 (64–74)	0.257
Alb	40 (36–42.25)	40 (36.25–43)	0.206
BNP	103 (44–387)	56 (26.5–187.5)	0.013
Troponin I	25.4 (7.4–284.1)	15.2 (6–110.8)	0.206
CK	58 (43.5–103.5)	77 (44–115)	0.446
AST	19 (15.5–25.5)	18 (15–24)	0.214

*Note:* HbA1c, glycated hemoglobin (normal range: 3.9–6.1%); CRP, C‐reactive protein (normal range: 0–5 mg/L), TP, total protein (normal range: 66–83 g/L); Alb, albumin (normal range: 39–50 g/L); BNP, brain natriuretic peptide (normal range: 10–100 pg/mL); troponin I (normal range: 1.9–15.6 ng/L); CK, creatine kinase (normal range: 45–200 IU/L); and AST, aspartate aminotransferase (normal range: 5–34 IU/L).

^∗^Mann–Whitney U test.

## 3. Results

### 3.1. Demographic Data

Table 1 presents the demographic data for the CHD and CHD–DED groups. The age shows no statistically significant difference between the CHD and CHD–DED groups, with a *p* value of  = 0.260. However, BMI differed significantly between the CHD and CHD–DED groups (*p* value < 0.006). Additionally, the BMI was higher than the normal range in both groups, suggesting a possible confounding variable for future investigations. There was no statistically significant difference between genders, with a *p* value of 0.119; however, the females were less in both groups.

### 3.2. Laboratory Findings

Table 2 illustrates the laboratory findings of the CHD and CHD–DED groups. Examination of inflammation‐related biomarkers showed a significant difference in HbA1c levels between the CHD group and the CHD–DED group (*p* value = 0.039). In contrast, there was no statistically significant difference in CRP levels between the CHD group and the CHD–DED group (*p* value = 0.206).

The analysis of protein biomarkers found no statistically significant differences in TP and Alb levels between the CHD group and the CHD–DED group (*p* values = 0.257 and 0.234, respectively).

The cardiac biomarkers for the CHD group and the CHD–DED group showed a statistically significant difference in BNP levels, *p* value = 0.013. However, troponin I, CK, and AST did not show statistically significant differences between the groups (*p* values = 0.206, 0.446, and 0.214, respectively).

### 3.3. Spearman’s Correlation Analyses of Inflammatory and Blood Biomarkers

Table 3illustrates the correlation within the CHD–DED group, highlighting statistically significant differences between the biomarkers; a strong positive correlation is observed between Alb and TP (*r* = 0.62, *p* value < 0.001), along with moderate negative correlations between BNP and Alb (*r* = −0.40, *p* value < 0.001).

**TABLE 3:** Spearman’s correlation coefficient and confidence intervals between BNP, Alb, TP, and CRP of CHD–DED.

Variable comparison	Groups	*r*	CI lower	CI upper	*p* value
BNP versus Alb	CHD–DED	−0.48	−0.65	−0.26	< 0.001
	CHD	−0.40	−0.54	−0.24	< 0.001

BNP versus TP	CHD–DED	−0.30	−0.61	0.09	0.06
	CHD	−0.24	−0.40	−0.07	< 0.001

BNP versus CRP	CHD–DED	0.21	−0.11	0.49	0.09
	CHD	0.03	−0.18	0.24	0.38

Alb versus TP	CHD–DED	0.75	0.53	0.87	< 0.001
	CHD	0.62	0.50	0.72	< 0.001

Alb versus CRP	CHD–DED	−0.10	−0.39	0.20	0.25
	CHD	0.02	−0.20	0.23	0.44

TP versus CRP	CHD–DED	0.25	−0.25	0.64	0.15
	CHD	−0.09	−0.30	0.12	0.19

Note: correlation coefficient (*r*).

Abbreviation: CI, confidence interval.

Conversely, Table 3 presents the correlation within the CHD group, revealing statistically significant differences among the biomarkers. There exists a strong positive correlation between Alb and TP (*r* = 0.75, *p* value < 0.001), a moderate negative correlation between BNP and Alb (*r* = −0.48, *p* value < 0.001), and a weak negative correlation between BNP and TP (*r* = −0.24, *p* value < 0.001).

### 3.4. Partial Spearman’s Correlations Adjusted for Age and BMI

In Table 4, both groups maintained an inverse relationship between BNP and Alb, as seen in partial Spearman’s correlation analyses that accounted for age and BMI. A moderate negative correlation was found between BNP versus Alb in the CHD–DED group and the CHD group (*p* value = 0.0297 and *p* value < 0.0001, respectively).

**TABLE 4:** Partial Spearman correlations adjusted for age and BMI.

Variable comparison	Group	^∗^Partial Spearman r	*p* value
BNP versus Alb	CHD–DED	−0.4536	0.0297
	CHD	−0.3689	< 0.0001

BNP versus TP	CHD–DED	−0.2391	0.2718
	CHD	−0.2210	0.0097

BNP versus CRP	CHD–DED	0.2396	0.4086
	CHD	0.3099	0.0025

Alb versus TP	CHD–DED	0.6731	0.0002
	CHD	0.6124	< 0.0001

*Note:* correlation coefficient (r).

Abbreviation: CI, confidence interval.

^∗^Partial correlations were calculated using Spearman correlations of regression residuals after adjustment for age and BMI.

Following adjustment, BNP versus TP showed a weak negative correlation in both groups, but it was not statistically significant in CHD–DED (*p* value = 0.27), whereas in the CHD group, it was statistically significant (*p* value = 0.0097). A positive correlation between BNP and CRP remained in both groups postadjustment, but it was statistically significant in CHD (*p* value = 0.0025), whereas in CHD–DED, it was not statistically significant (*p* value = 0.41).

In line with the unadjusted findings, Alb versus TP showed a strong positive correlation in both groups after adjusting for age and BMI (CHD: *p* value < 0.0001; CHD–DED: *p* value = 0.0002).

## 4. Discussion

This study investigated the level of natriuretic peptides and inflammatory biomarkers in patients with CHD–DED compared to those with CHD alone. The demographic study revealed that the mean age of the CHD–DED patients was elderly years. This result supports previous findings that CHD and DED are age‐dependent and related to the elderly [27, 28]. Furthermore, the male was higher than the female in both groups. This result is in line with previous studies on gender differences in CHD patients, which showed that prevalence rates are greater in males [29]. Conversely, DED is more common among females, as shown in previous research [4]. This research showed that BMI is statistically significant, with CHD–DED patients having higher BMI than normal. This aligns with earlier studies linking high BMI to CHD [30]. However, DED and a high BMI are inversely related [31]. Among the study’s findings, the demographic data in the CHD group were supported by previous research that found similar results to our study [27, 29, 30, 32, 33]. However, the demographic data, specifically the BMI and gender, in the CHD–DED group contradicted the data collected by previous studies [34, 35]. This study found that demographic data are more influenced by CHD comorbidity than by DED in the CHD–DED group.

The laboratory findings in this study found that CHD patients had mean BNP levels that were higher than those of CHD–DED patients; nevertheless, both groups demonstrated elevated BNP levels, indicating significant heart damage. This study’s findings showed that BNP levels were elevated in CHD patients, having a mean of 458.2, which is consistent with previous research and supports the idea that BNP is a robust biomarker for heart failure diagnosis and prognosis [36]. High BNP levels may indicate underlying heart damage conditions contributing to cardiovascular stress [37]. A previous study indicated that BNP is linked to inflammation and recommended testing it in other inflammatory conditions beyond the traditional focus on cardiac dysfunction conditions [38]. Additionally, a study indicates that BNP levels help distinguish proliferative retinopathy from nonproliferative retinopathy [39].

To our knowledge, no studies have explored the levels of BNP in DED patients, and our data can neither support nor contradict this. A potential interpretation of the lower BNP levels is that they might be linked with the development of DED.

The CHD–DED group showed greater mean HbA1c and CRP values compared to the CHD group. Nevertheless, both groups demonstrated elevated CRP levels, indicating significant inflammation. High CRP levels may indicate underlying inflammatory conditions contributing to cardiovascular stress [40]. Additionally, higher HbA1c levels typically indicate poor glycemic control, which can lead to both microvascular and macrovascular complications, potentially worsening CHD. Ultimately, this can exacerbate systemic inflammation and foster the development of DED through mechanisms such as oxidative stress, heightened immune responses, and ocular surface inflammation [12, 13].

The CHD group had higher TP and lower serum Alb levels than the CHD–DED group. Nevertheless, both groups demonstrated normal ranges of TP and Alb levels. This is a novel investigation; to our knowledge, it has not been explored. This finding indicates that TP and Alb levels are unreliable indicators of ocular complications in patients with CHD. Furthermore, it is essential to note that both Alb and TP levels are within the normal range, which makes them less helpful in identifying ocular manifestations. Thus, Alb and TP are not indicators for assessing ocular health in CHD–DED patients. This could be due to the complex array of medications and a unique interplay between CHD and DED.

Moreover, the mean CRP levels were more elevated among the CHD–DED patients compared to the CHD group. This finding contradicts a similar study that found no significant difference in mean CRP levels in patients with DLP and DLP‐DED [41]. This may suggest that DED is caused by fundamental processes that differ in CHD from DLP.

In addition, mean troponin I levels were severely elevated in both groups, with the CHD group showing higher mean levels than CHD–DED group. Furthermore, the mean of CK levels was within the normal range among both groups. Additionally, Troponin I is a crucial marker for diagnosing and measuring the severity of cardiac injury in CHD patients. The higher mean Troponin I levels in CHD patients indicate a recent or older Myocardial infarction [42]. Furthermore, we know that CK is highly concentrated in certain organs, including the heart, skeletal muscles, and brain. High levels of CK in CHD patients may indicate myocardial stress.

The CHD group had lower AST levels than the CHD–DED group. Nevertheless, both groups demonstrated normal ranges of AST levels. This is a novel investigation; to our knowledge, it has not been explored. This finding indicates that the AST level is an unreliable indicator of ocular complications in patients with CHD. The serum AST levels might not be related to CHD patients with ocular complications. The previous study revealed that the patients with DED had lower AST levels than non‐DED patients [43]. However, the CHD demonstrates a lower AST level than CHD–DED, our study did not reveal the same tendencies with AST levels between CHD and CHD–DED groups, which has not yet been reported.

Moreover, this study analyzed the correlation of laboratory findings for special test BNP and routine tests, Alb, TP, and CRP levels in patients between CHD and CHD–DED groups. The correlation results indicate that BNP is similar to the other biomarkers in both groups, except for a moderate positive correlation between BNP and CRP in CHD–DED. BNP and CRP are recognized biomarkers of cardiovascular disease, with CRP serving as a nonspecific marker of CHD; it has also been linked to adverse cardiovascular events, including DED [44]. The study recommends utilizing CRP to monitor and mitigate the risk of ocular complications in CHD for enhanced prognosis and diagnosis of DED. The presence of the natriuretic peptide system in the lacrimal gland regulates ocular surface homeostasis, and its disruption by systemic inflammation compromises this balance, a factor linked to DED symptoms [45].

Additionally, the study discovered the correlation in CRP versus TP was a weak positive correlation in the CHD group and a moderate negative correlation in CHD–DED, which were opposite in CRP versus Alb in both groups. This implies that increased CRP levels are linked to diseases and need more attention for CHD patients with ocular complications. The CHD–DED had a negative correlation with CRP, which was explained in previous studies decreased serum Alb levels during inflammatory conditions in CHD patients and DED patients [14, 46]. Casas et al. reported elevated CRP levels in patients with CHD, and Chien et al. reported decreased albumin levels in these patients [47, 48]. These findings support our results in a significant negative correlation between CRP and Alb in CHD–DED patients. Additionally, our findings in the correlation between CRP and Alb opposite between CHD groups support a study by Yoshioka et al. that shows that Alb level changes in CHD patients depend on their health condition [49].

### 4.1. Limitations and Future Work

This study’s retrospective design restricts the sample size to data that meet the inclusion criteria. Furthermore, the statistical models used did not adjust for potential confounders such as polypharmacy, acute hydration status during blood sampling, or clinical DED severity indices, all of which could impact the relationship between biomarkers like BNP and CRP and DED risk. Additionally, residual confounding from the severity of underlying CHD or lifestyle factors cannot be completely eliminated. These unaccounted variables might influence circulating biomarker levels, such as BNP and CRP, and their correlation with DED risk. Despite these limitations, the findings provide an important real‐world exploratory baseline that should be validated in prospective, multicenter studies with comprehensive adjustments.

Further research with a larger, multicenter, prospective, longitudinal sample is necessary to validate the findings and investigate BNP’s role in other inflammatory conditions. Additionally, strictly controlled cohorts should be used to systematically adjust for key confounders such as polypharmacy, acute hydration status, and cardiovascular disease severity. Moreover, incorporating standardized ocular measurements is crucial for accurately linking circulating plasma BNP levels with localized clinical DED severity and potential natriuretic peptide receptor therapies. Since this study has not yet been reported, more research is needed to understand the clinical blood characteristics of CHD patients with ocular issues.

## 5. Conclusions

This case–control study examined blood biomarkers, specifically BNP, along with inflammatory biomarkers as potential predictors of DED disease in patients with CHD. The study identifies potential protein biomarkers for CHD–DED patients, with strengthened correlations between inflammatory biomarkers supporting the underlying etiology. These routinely measured biomarkers could serve as potential clinical indicators for early detection of DED in patients with CHD.

## Author Contributions

Amani Y. Alhalwani was the study’s principal investigator and the primary researcher who designed the study, supervised the data collection, and wrote and approved the final version of this article. All authors revised the article and critically analyzed its intellectual, research, and statistical contents. All authors were involved in drafting the article and the data collection, and Shatha M. Jambi was also involved in data analysis. All authors contributed to the article

## Funding

The author(s) reported no funding associated with the work featured in this article.

## Disclosure

All claims expressed in this article are solely those of the authors and do not necessarily represent those of their affiliated organizations or those of the publisher, the editors, or the reviewers. Any product that may be evaluated in this article or claimed by its manufacturer is not guaranteed or endorsed by the publisher. All authors approved the submitted version.

## Ethics Statement

The study obtained ethical approval from the institutional review board at King Abdullah International Medical Research Center (Jeddah, Saudi Arabia) with Reference No. SPJ24/002/7. This study did not require written informed consent to participate by national legislation and institutional requirements.

## Conflicts of Interest

The authors declare no conflicts of interest.

## Data Availability

The data that support the findings of this study are available from the corresponding author upon reasonable request.
